# Development and external validation of a nomogram to predict survival and guide surgical strategy for colorectal cancer with synchronous liver and lung metastases: a SEER-based analysis

**DOI:** 10.3389/fonc.2026.1915006

**Published:** 2026-09-25

**Authors:** Hongqing Ma, Jianfeng Zhang, Qingyu Meng, Zesong Meng, Weijing Li, Chen Liu, Guiying Wang

**Affiliations:** 1 The Second Department of Surgery, The Fourth Hospital of Hebei Medical University, Shijiazhuang, China; 2 Hematology Department, The Fourth Hospital of Hebei Medical University, Shijiazhuang, China; 3 Department of General Surgery, The Second Hospital of Hebei Medical University, Shijiazhuang, China; 4 Hebei Key Laboratory of Etiology Tracing and Individualized Diagnosis and Treatment for Digestive system carcinoma, Shijiazhuang, China

**Keywords:** colorectal cancer, nomogram, overall survival, primary tumor resection, risk stratification, SEER database, synchronous liver and lung metastases

## Abstract

Surgical management of colorectal cancer with synchronous liver and lung metastases (CRC-SLLM) remains challenging because of marked clinical heterogeneity, and reliable tools for individualized risk assessment are lacking. We aimed to develop and externally validate a nomogram for overall survival (OS) prediction and to explore its potential value in guiding selection for primary tumor resection (PTR). Patients diagnosed with CRC-SLLM between 2010 and 2015 were identified from the Surveillance, Epidemiology, and End Results (SEER) database and randomly assigned to a training cohort (n = 808) and an internal validation cohort (n = 345) in a 7:3 ratio, whereas external validation was performed using 65 patients from the Fourth Hospital of Hebei Medical University, China. After feature selection with least absolute shrinkage and selection operator (LASSO) regression, a multivariable Cox-based nomogram was constructed, and its performance was assessed using time-dependent receiver operating characteristic curves, calibration plots, decision curve analysis (DCA), and risk stratification. The final nomogram incorporated nine predictors: age, primary tumor site, histological grade, tumor size, N stage, bone metastasis, carcinoembryonic antigen status, metastasectomy, and chemotherapy. It showed stable discrimination across the training, internal validation, and external validation cohorts, with 12-month areas under the curve (AUCs) of 0.791, 0.714, and 0.778 and 24-month AUCs of 0.742, 0.740, and 0.730, respectively; calibration was good in all cohorts, and DCA supported potential clinical utility. Nomogram-based risk stratification separated patients into prognostically distinct low- and high-risk groups, and an exploratory analysis indicated that the survival benefit of PTR was largely confined to low-risk patients. This externally validated nomogram provides individualized survival prediction and risk stratification for patients with CRC-SLLM and may help identify those more likely to benefit from PTR, although this finding remains exploratory and warrants confirmation in prospective multicenter studies.

## 1 Introduction

Colorectal cancer (CRC) ranks among the most prevalent malignancies globally and constitutes a major contributor to cancer mortality (1). At initial diagnosis, distant metastases are identified in 20%–25% of patients with colorectal cancer, which constitutes the primary cause of treatment failure and death. While the liver and lungs are the most common sites of hematogenous spread, synchronous liver and lung metastases (SLLM) represent a unique and highly aggressive clinical phenotype, accounting for roughly 3% to 5% of all CRC cases (2). Patients with SLLM generally exhibit profound tumor burdens, distinct molecular landscapes, and notably inferior survival outcomes compared to those with isolated organ metastasis, with a median overall survival (OS) historically hovering around 10 to 14 months if left untreated.

Despite the development of systemic therapies, the prognosis of SLLM exhibits substantial heterogeneity, complicating clinical decision-making (3). The American Joint Committee on Cancer (AJCC) Tumor-Node-Metastasis (TNM) staging system remains the gold standard for CRC prognostication; however, it classifies all SLLM patients into a homogeneous “M1b/M1c” category (4). This uniform classification fails to account for the intricate interplay of demographic, clinicopathological, and therapeutic variables, thereby limiting its capacity to deliver precise, individualized risk stratification (5).

Moreover, the role of primary tumor resection (PTR) in patients with asymptomatic stage IV CRC remains a subject of intense global debate (6). Recent randomized controlled trials, such as the CAIRO4 and SYNCHRONOUS studies, have cast doubt on the routine application of PTR in unselected metastatic CRC patients, highlighting the risk of delaying critical systemic therapies (7, 8). However, whether highly selected subpopulations—particularly within the SLLM cohort—might still derive survival benefits from aggressive surgical interventions have not been clearly defined.

To address these critical knowledge gaps, the present study aimed to construct a robust prognostic nomogram utilizing a large, population-based cohort from the Surveillance, Epidemiology, and End Results (SEER) database. Importantly, we sought to externally validate this model using an independent cohort from a Chinese tertiary cancer center and to utilize the nomogram-derived risk stratification system to identify the specific subset of SLLM patients who could truly benefit from primary tumor resection, thereby optimizing personalized surgical strategies.

## 2 Materials and methods

### 2.1 Study design and data sources

This retrospective, population-based cohort study incorporated an independent real-world external validation. The primary cohort was derived from the Surveillance, Epidemiology, and End Results (SEER) 17 registries database (2000–2022), and data extraction was performed using SEER*Stat software (version 9.0.42). The SEER program provides de-identified, publicly available data; accordingly, this component of the study was exempt from informed consent and Institutional Review Board (IRB) approval.

For external validation, a separate clinical cohort was retrospectively assembled from the Fourth Hospital of Hebei Medical University, China. This institution is a large tertiary hospital with one of the leading colorectal surgery departments in northern China, serving a broad patient population across multiple provinces, thereby ensuring both regional representativeness and clinical diversity. The external validation protocol was approved by the Ethics Committee of the Fourth Hospital of Hebei Medical University (IRB Approval Number: 2026KY104-01). This study was reported in accordance with the Strengthening the Reporting of Cohort Studies in Surgery (STROCSS) criteria (9) and the Transparent Reporting of a multivariable prediction model for Individual Prognosis or Diagnosis (TRIPOD) statement (10).

### 2.2 Patient selection

Patients with primary colorectal cancer (CRC) were identified using the International Classification of Diseases for Oncology, Third Edition (ICD-O-3) site codes C18.0–C20.9 (colon and rectum). Histological behavior was restricted to malignant tumors (ICD-O-3 behavior code = ‘Malignant’). Because detailed site-specific metastasis information was unavailable in SEER prior to 2010, the study period was confined to 2010–2015. Synchronous liver and lung metastases (SLLM) were defined as the concurrent presence of hepatic and pulmonary metastases documented at the time of initial CRC diagnosis, ascertained through the SEER variables “Combined Mets at DX-liver” and “Combined Mets at DX-lung.”

The inclusion criteria were as follows: (1) pathologically confirmed primary CRC; (2) synchronous liver and lung metastases at initial diagnosis; and (3) active follow-up with complete survival data. The exclusion criteria were: (1) unknown TNM stage; (2) unclear survival time or survival ≤1 month, to minimize the confounding effect of perioperative mortality unrelated to tumor progression; and (3) missing critical clinical or pathological variables.

After applying these criteria, 1,153 eligible patients from the SEER database were included. These patients were randomly allocated in a 7:3 ratio to a training cohort (n = 808) and an internal validation cohort (n = 345) for model development and internal validation, respectively. In parallel, 65 patients with CRC-SLLM treated at the Fourth Hospital of Hebei Medical University constituted the external validation cohort. The inclusion and exclusion criteria for the external cohort were identical to those applied to the SEER cohort to ensure population comparability. The study design is illustrated in **Figure 1**.

**Figure 1:**
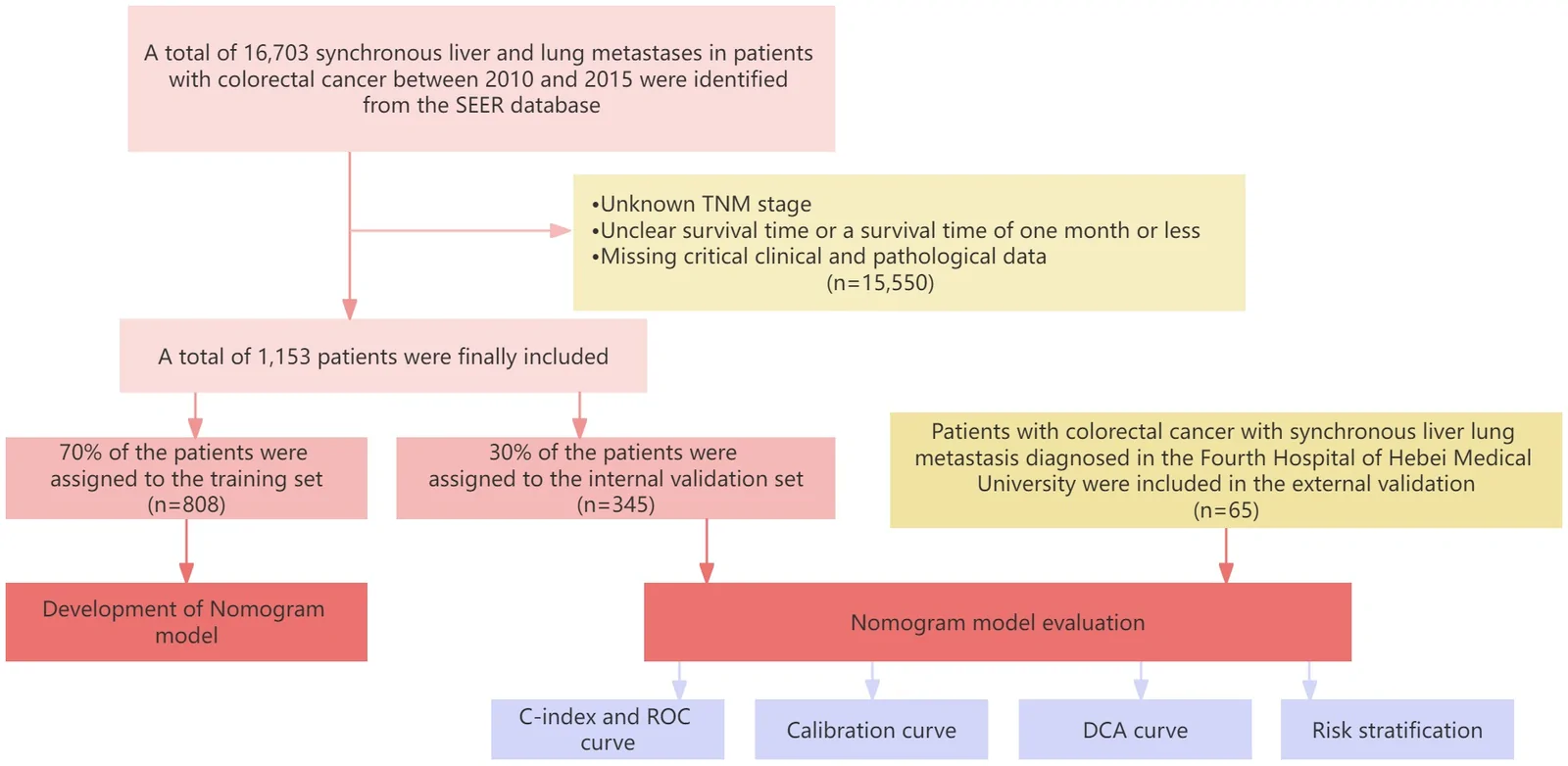
Study flowchart showing patient selection, cohort allocation, nomogram construction, and external validation.

### 2.3 Variable definition and selection

Fourteen candidate clinicopathological variables were extracted and predefined for model construction. Variables were categorized into demographic and tumor-related domains. The demographic domain comprised age, sex, and marital status, with age stratified as <60, 60–80, and >80 years. Tumor-related factors included the primary tumor site (right-sided colon, left-sided colon, and rectum), T stage, N stage, histological grade (Grade I, II, III, and IV), tumor size (≤5 cm and >5 cm), and preoperative carcinoembryonic antigen (CEA) level (negative and positive). Metastatic burden was characterized by bone metastasis (yes/no) and brain metastasis (yes/no). Treatment variables included systemic chemotherapy (yes/no/unknown), radiotherapy (yes/no), and metastasectomy (yes/no). Primary tumor resection (PTR) was not included as a candidate predictor but was reserved for the subsequent risk-stratified survival analysis.

TNM staging was classified according to the 7th edition of the American Joint Committee on Cancer (AJCC) staging system, consistent with the SEER coding scheme during the study period (2010–2015). For the external validation cohort, all variables were extracted from electronic medical records, with definitions maintained consistent with the SEER dataset. To preserve statistical power and reflect real-world clinical practice, variables with missing values (e.g., CEA status) were retained as a separate “Unknown” category rather than excluded.

### 2.4 Nomogram development and validation

Clinically established cut-off points were applied to transform continuous variables into categorical variables. Categorical data were summarized as frequencies and percentages. Overall survival (OS) was defined as the interval from the date of initial diagnosis to the date of death from any cause or last follow-up. Continuous predictors were dichotomized primarily to preserve the clinical interpretability and bedside usability of the nomogram, using cut-offs with established clinical relevance (age 60 and 80 years; tumor size 5 cm) rather than data-driven optimal cut-points; the potential impact of this approach on statistical power and calibration is addressed in the Discussion.

Univariate Cox proportional hazards regression was first performed in the training cohort to screen variables significantly associated with OS (P < 0.05). To address potential overfitting and optimize variable selection, least absolute shrinkage and selection operator (LASSO) Cox regression was subsequently applied. Variables retained by LASSO were then entered into a multivariable Cox regression model to identify independent prognostic factors. Prior to multivariable analysis, multicollinearity among candidate variables was evaluated using the variance inflation factor (VIF) and tolerance. The proportional hazards assumption was assessed using Schoenfeld residuals. Independent prognostic factors identified in the final multivariable model were used to construct a nomogram predicting OS at specified time points.

Variable selection was based on the one-standard-error criterion from tenfold cross-validation (λ1se = 0.112), and all candidate variables were entered into both the LASSO and the multivariable Cox models as categorical variables using the clinically established cut-offs described in Section 2.3. In the training cohort, 787 deaths occurred among the 808 patients; the final nine-predictor model corresponded to 14 degrees of freedom under categorical coding, yielding approximately 56 events per variable, which far exceeds the conventional minimum of 10 recommended for stable estimation of a multivariable Cox model. The external cohort (n = 65) was used solely for external validation and was not intended to support definitive subgroup inference.

The predictive performance of the nomogram was evaluated across the training, internal validation, and external validation cohorts with respect to discrimination and calibration. Discrimination was quantified by the concordance index (C-index) and the area under the time-dependent receiver operating characteristic (ROC) curve (AUC). Calibration was assessed using calibration plots generated from 1,000 bootstrap resamples, comparing nomogram-predicted survival probabilities against observed outcomes. Decision curve analysis (DCA) was performed to evaluate the clinical net benefit of the nomogram across a range of threshold probabilities.

### 2.5 Statistical analysis

Categorical variables were summarized using counts and proportions. Overall survival was defined as the interval between the date of initial diagnosis and either death from any cause or the last recorded follow-up. The multivariable Cox regression model, LASSO Cox regression, Kaplan–Meier method, log-rank test, time-dependent ROC, calibration plots, and DCA were systematically executed.

All statistical analyses and graphical representations were performed using R software version 4.2.2 (http://www.R-project.org; The R Foundation, Vienna, Austria) and the Free Statistics software version 2.3 (Beijing Free Clinical Medical Technology Co., Ltd, Beijing, China). All statistical tests were two-sided, and a P-value of < 0.05 was considered to indicate statistical significance.

## 3 Results

### 3.1 Patient characteristics

From the Surveillance, Epidemiology, and End Results (SEER) database, 16,703 patients diagnosed with colorectal cancer and synchronous liver–lung metastases (SLLM) between 2010 and 2015 were initially retrieved. A total of 15,550 patients were subsequently excluded owing to unknown TNM stage, ambiguous or absent survival data, survival duration ≤1 month, or incomplete clinicopathological records, leaving 1,153 patients eligible for the final analysis. These patients were randomly divided into a training cohort (n = 808, 70%) and an internal validation cohort (n = 345, 30%). Separately, 65 consecutive SLLM patients treated at the Fourth Hospital of Hebei Medical University constituted the external validation cohort (**Figure 1**). Baseline demographic, clinicopathological, and treatment characteristics of the three cohorts are summarized in **Table 1**.

**Table 1:** Baseline demographic and clinicopathological characteristics of colorectal cancer with synchronous liver and lung metastases across the training, internal validation, and external validation cohorts.

Variable	Training cohort	Internal validation cohort	External validation cohort
N (%)	n = 808	n = 345	n = 65
Age, n (%)			
<60	358 (44.3)	156 (45.2)	15 (23.1)
60-80	366 (45.3)	163 (47.2)	47 (72.3)
>80	84 (10.4)	26 (7.5)	3 (4.6)
Sex, n (%)			
male	434 (53.7)	200 (58)	34 (52.3)
female	374 (46.3)	145 (42)	31 (47.7)
Tumor size, n (%)			
≤5cm	325 (40.2)	135 (39.1)	16 (24.6)
>5cm	483 (59.8)	210 (60.9)	49 (75.4)
Grade, n (%)			
I	37 (4.6)	18 (5.2)	2(3.1)
II	595 (73.6)	251 (72.8)	54 (83.1)
III	149 (18.4)	61 (17.7)	9 (13.8)
IV	27 (3.3)	15 (4.3)	0(0)
Primary site, n (%)			
Right-sided colon	313 (38.7)	116 (33.6)	16 (24.6)
Left-sided colon	321 (39.7)	145 (42)	16 (24.6)
Rectum	174 (21.5)	84 (24.3)	33 (50.8)
T Stage, n (%)			
T1	94 (11.6)	46 (13.3)	0 (0)
T2	26 (3.2)	10 (2.9)	1 (1.5)
T3	408 (50.5)	157 (45.5)	38 (58.5)
T4	280 (34.7)	132 (38.3)	26 (40.0)
N Stage, n (%)			
N0	177 (21.9)	93 (27)	5 (7.7)
N1	340 (42.1)	130 (37.7)	18 (27.7)
N2	291 (36)	122 (35.4)	42 (64.6)
Bone metastasis, n (%)			
NO	738 (91.3)	314 (91)	60 (92.3)
YES	70 (8.7)	31 (9)	5 (7.7)
Brain metastasis,n (%)			
NO	795 (98.4)	338 (98)	65 (100)
YES	13 (1.6)	7 (2)	0(0)
Primary resection, n (%)			
NO	510 (63.1)	236 (68.4)	43 (66.2)
YES	298 (36.9)	109 (31.6)	22 (33.8)
Metastasectomy, n (%)			
NO	670 (82.9)	297 (86.1)	60(92.3)
YES	138 (17.1)	48 (13.9)	5(7.7)
Radiation, n (%)			
NO	690 (85.4)	291 (84.3)	60 (92.3)
YES	118 (14.6)	54 (15.7)	5 (7.7)
Chemotherapy, n (%)			
NO	187 (23.1)	73 (21.2)	16 (24.6)
YES	621 (76.9)	272 (78.8)	49 (75.4)
CEA, n (%)			
Negative	74 (9.2)	37 (10.7)	5 (7.7)
Positive	734 (90.8)	308 (89.3)	60 (92.3)

### 3.2 Feature selection using LASSO regression

Fourteen candidate clinical and pathological variables were entered into a LASSO Cox regression model to identify the most informative predictors of OS. The coefficient profile plot illustrates the trajectory of each variable’s regression coefficient as the penalty parameter (λ) increases; coefficients of less contributory variables were progressively shrunk toward zero and eventually eliminated (**Figure 2a**). To determine the optimal λ, tenfold cross-validation was conducted; the resulting plot displays partial likelihood deviance as a function of log(λ), with two vertical dashed lines marking the λmin (minimum deviance) and λ1se (one standard error above the minimum) criteria, respectively (**Figure 2a**). At λ1se (λ = 0.112), nine variables retained non-zero coefficients and were carried forward for model construction: age at diagnosis, primary tumor site, histological differentiation grade, tumor size, N stage, bone metastasis, serum CEA status, metastasectomy, and chemotherapy receipt. The remaining five candidate variables — sex, T stage, brain metastasis, radiotherapy, and marital status — were shrunk to zero at λ1se and were therefore excluded from the final model.

**Figure 2:**
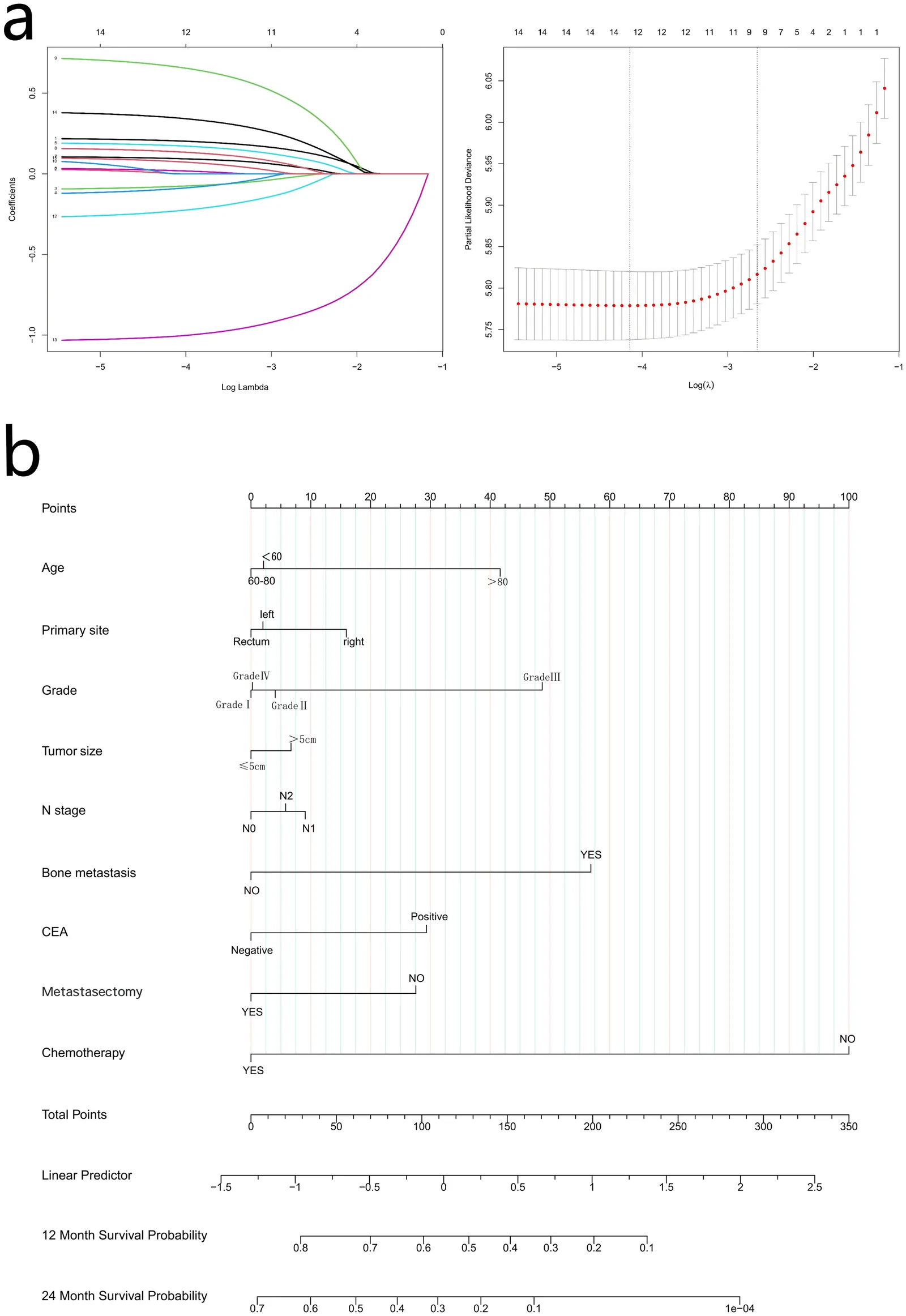
LASSO-based feature selection and prognostic nomogram for overall survival. **(a)** Coefficient profiles of candidate variables in the LASSO Cox regression model and tenfold cross-validation plot used to determine the optimal value of λ. **(b)** Nomogram incorporating the selected prognostic factors for individualized prediction of 12-month and 24-month overall survival in patients with colorectal cancer with synchronous liver and lung metastases.

### 3.3 Multivariable Cox regression and nomogram construction

A multivariable Cox proportional hazards model was constructed using the nine variables selected through LASSO. All nine factors retained independent prognostic significance after mutual adjustment (**Table 2**). Older age (≥80 years vs. <60 years), right-sided primary tumor location, advanced nodal stage, poor differentiation (Grade III), tumor size >5 cm, positive CEA, presence of bone metastasis, and absence of metastasectomy were each associated with a significantly elevated hazard of death. Chemotherapy, by contrast, emerged as a protective factor with a significantly reduced hazard ratio.

**Table 2:** Multivariable Cox proportional hazards analysis of overall survival in the training cohort. .

Variable	Category	HR (95% CI)	P value
Age	<60 y	Reference	—
	60–80 y	1.11 (0.98–1.26)	0.103
	>80 y	1.83 (1.47–2.28)	<0.001
Primary tumor site	Right-sided colon	Reference	—
	Left-sided colon	0.73 (0.61–0.86)	<0.001
	Rectum	1.03 (0.83–1.26)	0.810
Histological grade	Grade I	Reference	—
	Grade II	1.11 (0.83–1.47)	0.489
	Grade III	1.55 (1.13–2.13)	0.006
	Grade IV	1.30 (0.85–1.96)	0.223
Tumor size	≤5 cm	Reference	—
	>5 cm	1.18 (1.05–1.34)	0.007
N stage	N0	Reference	—
	N1	1.13 (0.96–1.33)	0.152
	N2	1.29 (1.08–1.53)	0.005
Bone metastasis	No	Reference	—
	Yes	2.28 (1.84–2.82)	<0.001
CEA status	Negative	Reference	—
	Positive	1.48 (1.20–1.82)	<0.001
Metastasectomy	No	Reference	—
	Yes	0.77 (0.65–0.91)	0.002
Chemotherapy	No	Reference	—
	Yes	0.34 (0.29–0.39)	<0.001

HR, hazard ratio; CI, confidence interval; CEA, carcinoembryonic antigen. The reference category for primary tumor site is right-sided colon.

Multicollinearity among the nine predictors was minimal: the maximum generalized variance inflation factor (GVIF) was 1.23 (adjusted GVIF^1/(2·df) = 1.05), well below thresholds of concern. Assessment of the proportional hazards assumption using Schoenfeld residuals showed no significant departure for seven of the nine predictors (all P > 0.05); a time-varying effect was detected for chemotherapy (P < 0.001) and, to a lesser extent, age (P = 0.013), while the global test was also significant (χ2 = 41.85, df = 14, P < 0.001). The clinical implications of this finding are addressed in the Discussion.

A prognostic nomogram incorporating these nine independent predictors was then constructed to estimate individualized 12-month and 24-month OS probabilities for SLLM patients (**Figure 2b**). In practical use, the clinician locates each patient’s variable category on its respective axis, reads the corresponding point value from the top “Points” scale, and sums all individual scores to obtain a “Total Points” value. This total is then projected downward onto the survival probability axes to derive the predicted 12-month and 24-month OS.

### 3.4 Discriminative performance

Time-dependent receiver operating characteristic (ROC) curves were plotted, and the area under the curve (AUC) was calculated for each cohort at 12 and 24 months (**Figure 3**). In the training cohort, the AUC for 12-month OS was 0.791 (95% CI, 0.759–0.823) and for 24-month OS was 0.742 (95% CI, 0.708–0.777). The internal validation cohort yielded AUC values of 0.714 (95% CI, 0.659–0.768) and 0.740 (95% CI, 0.686–0.794) for 12-month and 24-month OS, respectively. Notably, when the model was applied to the geographically and ethnically independent external validation cohort, the AUC remained 0.778 (95% CI, 0.646–0.890) at 12 months and 0.730 (95% CI, 0.595–0.865) at 24 months. The preservation of discriminative accuracy across a US population–based registry and a Chinese single-institution dataset indicates robust transportability of the nomogram.

**Figure 3:**
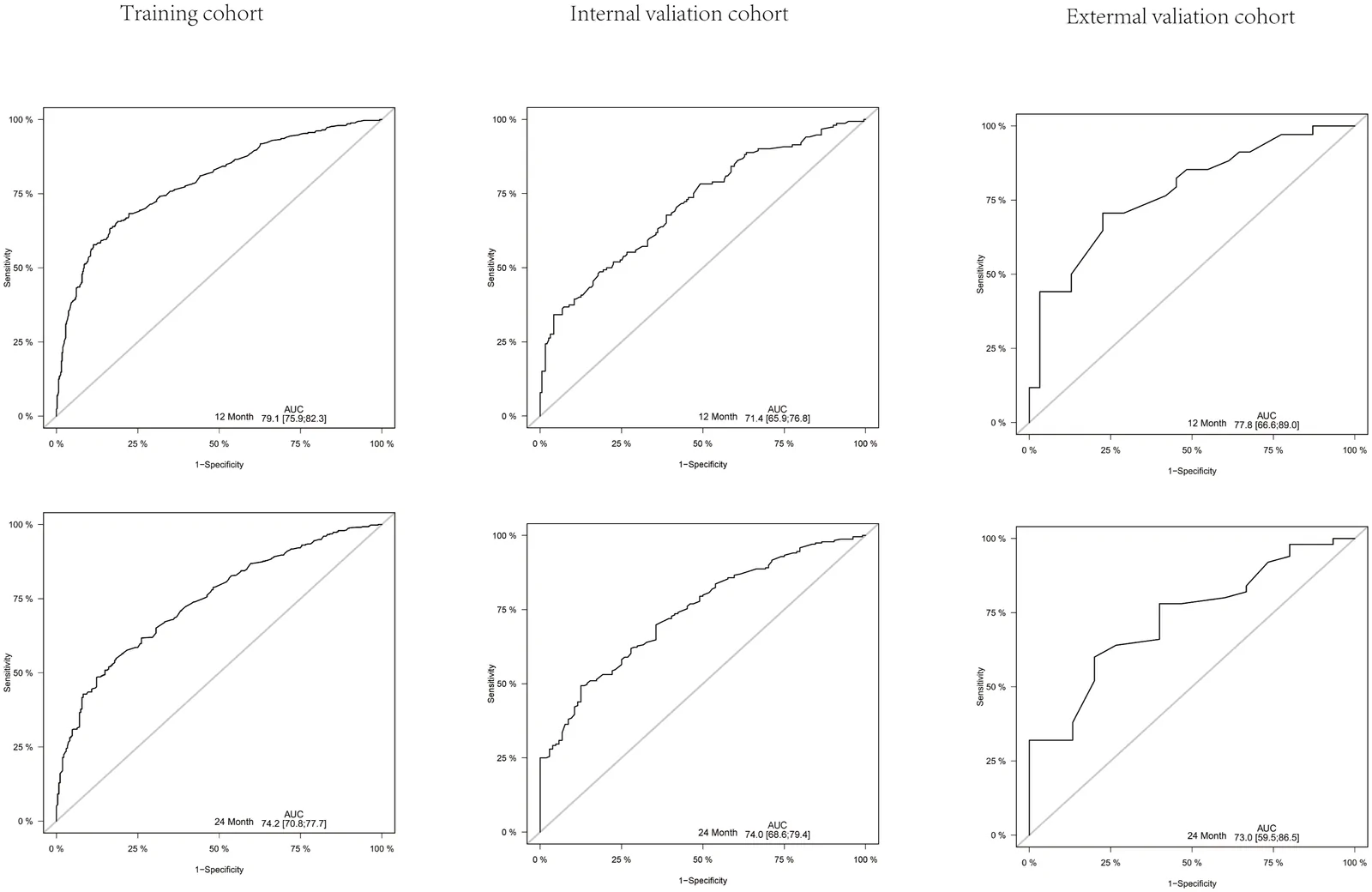
Discriminative performance of the nomogram assessed by time-dependent ROC analysis in the training, internal validation, and external validation cohorts.

To complement the time-dependent AUC, the overall concordance index (C-index) was also calculated. The C-index was 0.701 (95% CI, 0.681–0.720) in the training cohort, 0.660 (95% CI, 0.626–0.695) in the internal validation cohort, and 0.810 (95% CI, 0.745–0.875) in the external validation cohort. Internal validation using 500 bootstrap resamples yielded an optimism-corrected C-index of 0.693 and a calibration slope of 0.927, indicating minimal overfitting. As a time-integrated summary measure, the time-averaged AUC was 0.797 over 0–24 months and 0.769 over 0–60 months, consistent with the time-dependent AUC values reported above.

### 3.5 Calibration

**Figure 4** presents the calibration plots comparing nomogram-estimated survival probabilities with the corresponding observed outcomes at 12 and 24 months. In all three cohorts, the calibration plots lay in close proximity to the ideal 45-degree diagonal, reflecting a high degree of concordance between predicted and observed survival. No systematic pattern of over- or under-estimation was apparent, confirming that the nomogram generated reliable and unbiased survival estimates in both the derivation and validation settings.

**Figure 4:**
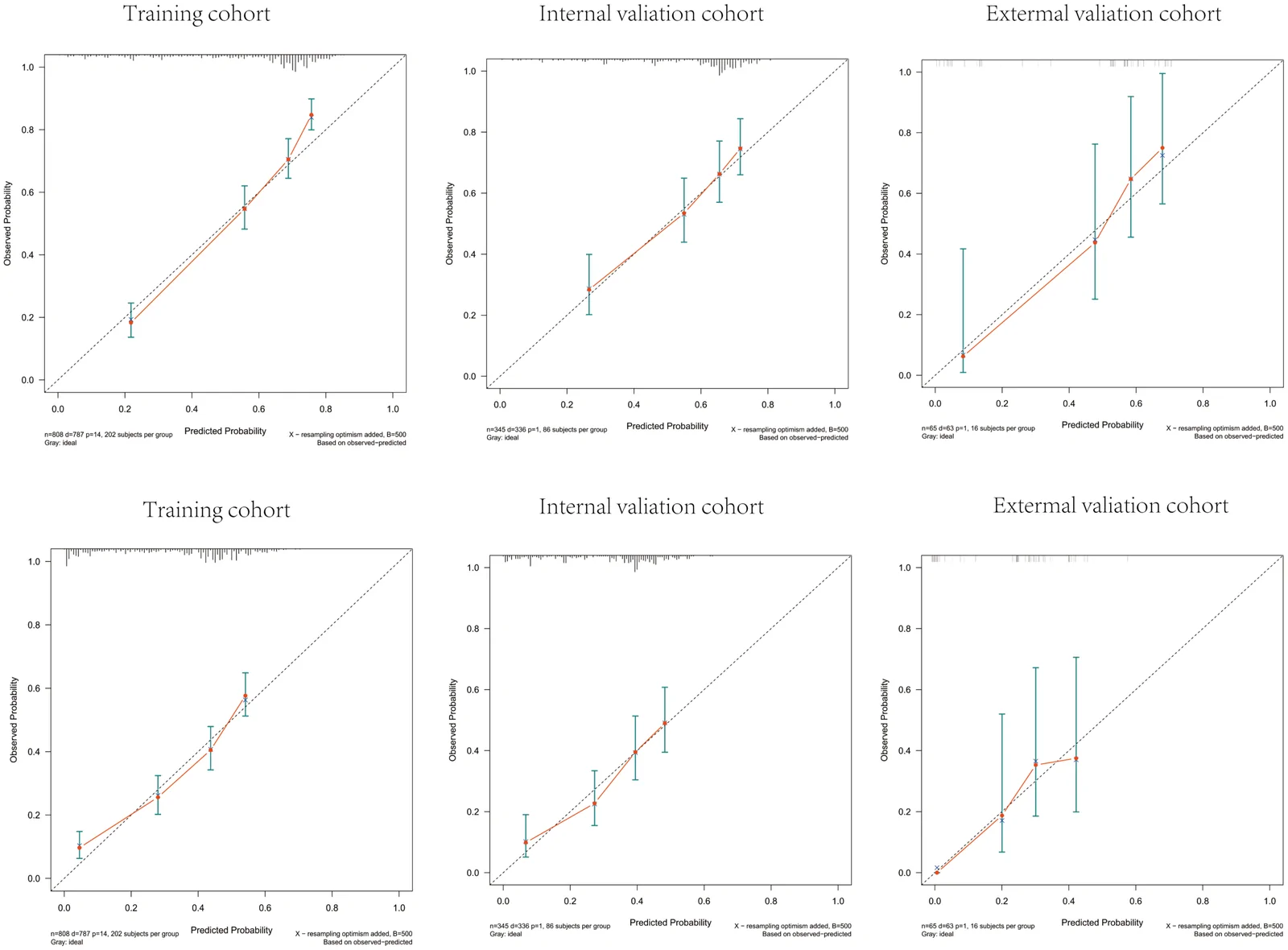
Calibration curves of the nomogram for predicting 12-month and 24-month overall survival in the training, internal validation, and external validation cohorts.

### 3.6 Clinical utility: decision curve analysis

Decision curve analysis was performed to assess the clinical utility of the nomogram (**Figure 5**). For 12-month OS, the nomogram showed a higher net benefit than the treat-all and treat-none strategies across an approximate threshold probability range of 27% to 88% in the training cohort, and similar findings were observed in the internal and external validation cohorts. For 24-month OS, the nomogram likewise demonstrated a favorable net benefit across a broad range of clinically relevant thresholds in all three cohorts. Taken together, these results suggest that the nomogram may offer additional clinical value for individualized decision-making; however, given the limited size of the external validation cohort (n = 65), these findings should be interpreted with appropriate caution and require confirmation in larger, prospective cohorts.

**Figure 5:**
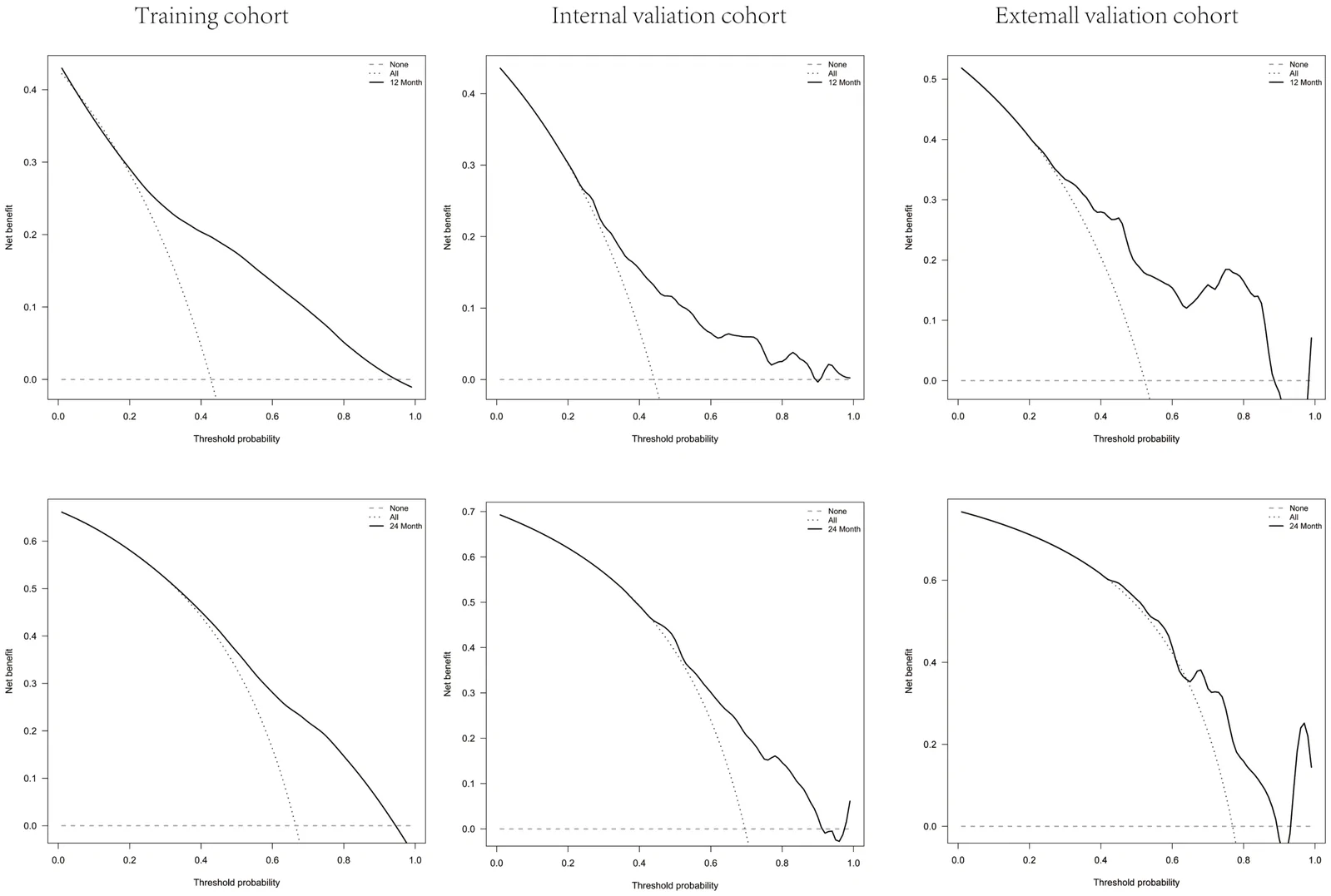
Decision curve analysis of the nomogram for predicting 12-month and 24-month overall survival in the training, internal validation, and external validation cohorts.

### 3.7 Risk stratification and survival analysis

Using the nomogram-predicted prognostic score in the training cohort, patients were dichotomized at the median score (−0.291) into a low-risk group (score ≤ −0.291) and a high-risk group (score > −0.291). The median-based cutoff derived from the training cohort was then applied identically to the internal and external validation cohorts to ensure consistency in risk classification across all three cohorts. In the training cohort, 404 patients were classified as low-risk and 404 as high-risk, and Kaplan–Meier analysis demonstrated a clear separation in survival between the two groups (log-rank P < 0.0001; **Figure 6a**). Comparable separation was observed in the internal validation cohort (low-risk, n = 186; high-risk, n = 159; log-rank P < 0.0001; **Figure 6b**) and in the external validation cohort (low-risk, n = 20; high-risk, n = 45; log-rank P = 0.021; **Figure 6c**). This threshold was pre-specified using only the training cohort data and was subsequently applied, without modification or re-optimization, to the internal and external validation cohorts, thereby avoiding threshold tuning on validation data.

**Figure 6:**
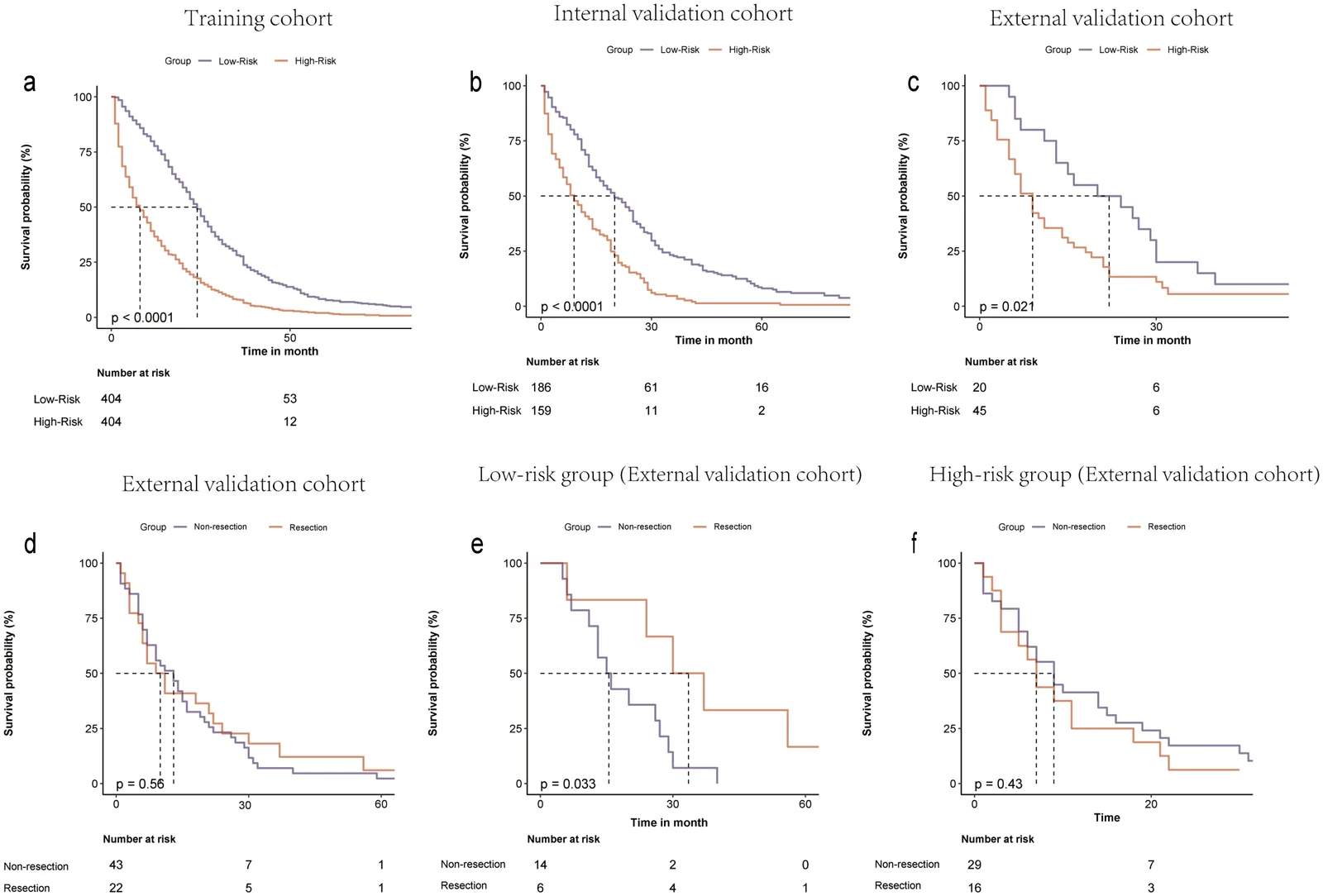
Kaplan–Meier survival analyses stratified by the nomogram-derived risk score and primary tumor resection (PTR) status. Patients were dichotomized into low- and high-risk groups using a nomogram cutoff of −0.291 derived from the training cohort. **(a)** Training cohort (n = 808). **(b)** Internal validation cohort (n = 345). **(c)** External validation cohort (n = 65). **(d)** PTR versus non-resection across the entire external validation cohort. **(e, f)** PTR versus non-resection within the low-risk **(e)** and high-risk **(f)** subgroups of the external validation cohort. Between-group comparisons were made with the log-rank test. Numbers at risk are listed below each curve.

We further examined the association between primary tumor resection (PTR) and survival in the external validation cohort. When the external cohort was analyzed as a whole, overall survival did not differ significantly between resected (n = 22) and non-resected patients (n = 43; log-rank P = 0.56; **Figure 6d**). After stratification by the nomogram-defined risk group, however, the pattern differed. Among low-risk patients, those who underwent PTR (n = 6) had significantly longer survival than those who did not (n = 14; log-rank P = 0.033; **Figure 6e**). By contrast, within the high-risk group, PTR (n = 16) conferred no significant survival advantage over non-resection (n = 29; log-rank P = 0.43; **Figure 6f**). These findings suggest that the survival benefit of PTR in CRC-SLLM is largely confined to patients identified as low-risk by the nomogram. The web-based calculator (https://hbykdxdsyy.shinyapps.io/SLLM_Nom_Cox_model/) enabled individualized risk prediction.

## 4 Discussion

In this study, we constructed a prognostic nomogram for colorectal cancer patients with synchronous liver and lung metastases (SLLM) using data from the SEER registry and validated it against an independent Chinese single-center cohort. Nine routinely available clinicopathological variables were incorporated into the model, which demonstrated stable discriminative accuracy, good calibration, and positive clinical net benefit across all three cohorts. Nomogram-based risk stratification successfully separated patients into prognostically distinct groups, and an exploratory subgroup analysis indicated that the survival advantage conferred by primary tumor resection (PTR) appeared to be confined to patients in the low-risk category. These observations position the nomogram as both a prognostic instrument and a potential decision-support tool for surgical planning in this difficult-to-manage population.

CRC-SLLM occupies a unique niche in the spectrum of metastatic disease. Patients with isolated liver or lung metastases can be assessed against established resectability criteria and benefit from mature, organ-specific treatment algorithms (11, 12). No comparable framework exists for those with concurrent hepatic and pulmonary involvement—a phenotype that, by definition, implies multi-compartmental hematogenous spread. The AJCC TNM system groups all such patients under M1b, yet the clinical reality within this single staging category is anything but homogeneous (13). A relatively fit patient in their fifties with a left-sided, well-differentiated primary and no extra-hepatopulmonary disease confronts a fundamentally different prognosis from an octogenarian with a right-sided, poorly differentiated tumor and concurrent skeletal involvement (14, 15). Conventional staging cannot capture these gradations. The nomogram developed here was designed to address precisely this shortcoming: by synthesizing multiple clinical parameters into one quantitative score, it offers finer prognostic resolution than stage grouping alone and may better inform the increasingly nuanced decisions that characterize modern multidisciplinary oncology.

The nine variables retained through LASSO selection and confirmed by multivariable Cox regression map onto four clinically intuitive dimensions. First, host condition: older age was independently associated with worse survival, reflecting the cumulative impact of diminished organ reserve, competing comorbidities, and reduced eligibility for dose-intensive systemic regimens in elderly patients with advanced CRC (16, 17). Second, tumor aggressiveness: right-sided primary location, higher N categories, poor histological differentiation, and elevated serum CEA each point toward a more biologically unfavorable primary tumor. The prognostic inferiority of right-sided CRC in the metastatic setting is well recognized and is thought to relate to its distinct molecular underpinnings—including a higher prevalence of BRAF mutations, CMS1 consensus subtype, and inferior responsiveness to anti-EGFR therapy (14, 15). Elevated CEA, meanwhile, remains one of the most robust serum surrogates of total tumor volume and proliferative activity in CRC and has been consistently linked to adverse outcomes across disease stages (18, 19). Third, metastatic extent: the retention of bone metastasis in the model deserves particular comment. Skeletal involvement in CRC is infrequent and almost invariably occurs in the setting of widespread dissemination; its presence therefore serves as a clinical marker of high systemic disease burden beyond the hepatopulmonary compartment (20). Fourth, therapeutic opportunity: chemotherapy receipt emerged as a protective factor, consistent with the established survival benefit of systemic therapy in metastatic CRC (21). Metastasectomy likewise carried a favorable prognostic signal. While this likely reflects a degree of selection bias—patients referred for metastasectomy tend to have more limited, technically resectable disease and better performance status—it also aligns with the growing evidence that carefully selected patients with oligometastatic CRC can achieve meaningful long-term survival through locoregional consolidation (22). The coherent clinical rationale underlying each variable reinforces the face validity of the model.

A particular strength of this study is the multi-tiered validation strategy. Within the SEER-derived population, the training-cohort AUC values of 0.791 (12-month OS) and 0.742 (24-month OS) represent good discrimination for a model relying exclusively on standard clinical variables without molecular or genomic input. Internal validation produced comparable figures (0.714 and 0.740, respectively), ruling out gross overfitting. The more stringent test, however, came from applying the model to a geographically, ethnically, and systemically different cohort: the 65-patient external dataset from a Chinese academic hospital. Here the AUC was 0.778 at 12 months and 0.730 at 24 months, with calibration curves remaining close to the 45-degree ideal. This degree of cross-population stability is notable because SEER-derived prognostic models are often criticized for their limited transportability, and performance degradation is the norm rather than the exception when such tools are tested outside the United States (23, 24). The retained accuracy across these divergent clinical environments suggests that the prognostic factors identified capture aspects of disease biology that transcend population-specific confounders. Decision curve analysis added a further practical dimension: across a wide range of threshold probabilities, the nomogram delivered a higher net benefit than either treating all patients or withholding treatment entirely, indicating that its use in clinical workflows would improve decision quality relative to default strategies.

From a surgical standpoint, the most provocative finding concerns the relationship between PTR and survival across risk strata. Whether to resect the primary tumor in patients with asymptomatic stage IV CRC has been debated for over a decade, and recent randomized evidence has shifted the consensus away from routine upfront resection. The CAIRO4 trial demonstrated no overall survival benefit for PTR in patients with synchronous unresectable metastases (7), and the SYNCHRONOUS study reached a similar conclusion (8). These results have tempered enthusiasm for routine PTR and redirected attention toward prompt initiation of systemic therapy. It should be noted, however, that these landmark trials enrolled broadly defined metastatic populations; neither was designed or powered to detect benefits within specific prognostic subsets.

Our exploratory analysis within the external validation cohort provides a preliminary but biologically coherent signal. Low-risk patients who underwent PTR survived significantly longer than those managed non-operatively (log-rank P = 0.033), whereas no such association was seen in the high-risk group (P = 0.43). We are cautious not to overstate these results: the subgroups were small (6 resected vs. 14 unresected in the low-risk stratum), and a retrospective comparison cannot account for the many unmeasured factors that drive surgical selection. Nevertheless, the direction of the finding is plausible. Patients with a favorable baseline profile are more likely to tolerate the physiological insult of surgery without compromising subsequent systemic treatment, and may derive additional benefit from removing a source of ongoing immunosuppressive signaling, obstructive complications, and continued metastatic seeding. In contrast, when disease biology is inherently aggressive and systemic burden is high, the therapeutic window consumed by operative recovery and convalescence may simply outweigh any oncological gain. This interpretation dovetails with the broader oncological principle that locoregional interventions yield diminishing returns as systemic disease dominance increases. We view these data as hypothesis-generating: they do not justify a change in current practice, but they do provide a rationale for future prospective evaluation of risk-stratified surgical strategies in SLLM.

The 2010–2015 study period warrants explanation. SEER has systematically recorded site-specific metastatic involvement (via the “Combined Mets at DX” variables) only since 2010, precluding reliable identification of synchronous liver and lung metastases before that year; the upper limit of 2015 was chosen to ensure sufficiently mature survival follow-up and to preserve consistency with the AJCC 7th edition TNM staging applied throughout this interval, thereby avoiding the staging discontinuity introduced by the AJCC 8th edition from 2018. We acknowledge that systemic therapy has since evolved, notably immune checkpoint inhibition for the small subset of patients with deficient mismatch repair/microsatellite instability–high tumors and broader biomarker-guided targeted therapy. Furthermore, the SEER database does not capture molecular markers such as RAS and BRAF mutation status or microsatellite instability (MSI) status, nor does it provide granular metastatic burden details (e.g., the number or size of hepatic and pulmonary lesions), all of which are increasingly recognized as important prognostic and predictive factors in metastatic colorectal cancer. Because our model relies on routinely available clinicopathological variables rather than treatment- or biomarker-specific data, it captures a stable baseline prognostic signal; nevertheless, it does not account for these newer therapies or molecular determinants, which may modify absolute survival. Reassuringly, the external validation was performed on a more contemporary institutional cohort (2015–2020), and the preserved discrimination suggests that the core prognostic structure remains applicable, although recalibration on contemporary population-based data will be valuable as longer follow-up accrues.

Baseline characteristics differed appreciably between the SEER and external cohorts, most notably in the proportion of rectal primaries (50.8% vs. 21.5%) and N2 disease (64.6% vs. 36.0%). These differences likely reflect distinct referral and surgical case-selection patterns between a population-based US registry and a single high-volume Chinese surgical center. Rather than undermining the model, the preservation of discrimination and calibration across these divergent case mixes argues for the robustness and transportability of the identified prognostic structure. Nonetheless, the single-center nature and modest size of the external cohort constrain broader generalizability, and validation across additional, more heterogeneous populations remains warranted.

Several limitations should be acknowledged. First, the external validation cohort was modest in size (n = 65), and the primary tumor resection subgroups were correspondingly small (e.g., 6 vs. 14 patients in the low-risk stratum); these analyses were therefore underpowered and should be regarded as exploratory and hypothesis-generating rather than confirmatory. Second, the retrospective design cannot exclude residual confounding, particularly selection effects influencing which patients underwent surgery. Third, the model is based on routinely available clinicopathological variables and does not incorporate molecular or genomic determinants. Fourth, continuous variables such as age and tumor size were dichotomized using clinically established cut-offs to enhance interpretability, which may have reduced statistical power and calibration relative to modelling these variables as continuous or spline terms. Fifth, although the majority of predictors satisfied the proportional hazards assumption, chemotherapy and, to a lesser extent, age showed evidence of time-varying effects; because the nomogram is intended to predict survival probabilities at fixed time horizons rather than to estimate constant hazard ratios, and because performance was evaluated using time-dependent and time-integrated discrimination measures that do not require strict proportionality, this deviation is unlikely to materially affect predictive utility, although it is acknowledged as a methodological limitation. Prospective, multicenter studies with larger cohorts are needed to confirm the risk-stratified surgical strategy suggested here before clinical implementation.

## 5 Conclusion

This study developed and externally validated a survival nomogram for patients with colorectal cancer and synchronous liver and lung metastases. Based on nine routinely available clinicopathological variables, the model demonstrated stable predictive performance across the SEER cohort and an independent Chinese external cohort. The nomogram-derived risk classification showed that primary tumor resection may improve survival in patients at low risk, but no comparable benefit was evident in the high-risk group. While this observation remains exploratory because of the limited size of the external cohort and the retrospective study design, it indicates that the model may aid in refining patient selection for primary tumor resection. Prospective multicenter validation is warranted before clinical implementation.

## Data Availability

The SEER datasets analyzed in this study are publicly available from the Surveillance, Epidemiology, and End Results Program of the U.S. National Cancer Institute (https://seer.cancer.gov/) upon completion of the standard SEER Research Data Agreement. The external validation dataset from the Fourth Hospital of Hebei Medical University contains identifiable patient information and is therefore not publicly available due to institutional policy and patient privacy regulations; de-identified data are available from the corresponding author (Guiying Wang, 13932186739@139.com) on reasonable request, subject to approval by the Ethics Committee of the Fourth Hospital of Hebei Medical University (IRB Approval Number: 2026KY104-01).
