# Supplementary material for: Development and external validation of a nomogram to predict survival and guide surgical strategy for colorectal cancer with synchronous liver and lung metastases: a SEER-based analysis
Source: Front Oncol. 2026 Sep 25;16:1915006. doi: 10.3389/fonc.2026.1915006 (PMC13649468; doi:10.3389/fonc.2026.1915006)
Supplement: Supplementary file 1 [file Table1.docx]

**TRIPOD+AI Checklist — Completed**

*Manuscript: Development and external validation of a nomogram to predict survival and guide surgical strategy for colorectal cancer with synchronous liver and lung metastases: a SEER-based analysis*

Locations are given by manuscript section/heading (robust to repagination). If the editor requires page numbers, append them from the final compiled PDF. "N/A" items are not applicable to this conventional regression-based prognostic model and are annotated with a brief rationale. Checklist source: Collins GS, Moons KGM, Dhiman P, et al. BMJ 2024;385:e078378.

| **Section / Topic** | **Item** | **Reported (location in manuscript) / Status** |
| --- | --- | --- |
| **TITLE** | | |
| Title | 1 | Reported — Title page. The title identifies the study as developing and externally validating a multivariable prediction (nomogram) model, the target population (CRC with synchronous liver and lung metastases), and the predicted outcome (survival). |
| **ABSTRACT** | | |
| Abstract | 2 | Reported — Abstract (single structured paragraph). Completed per TRIPOD+AI for Abstracts. |
| **INTRODUCTION** | | |
| Background | 3a | Reported — Introduction, paragraphs 1–2 (prognostic context of CRC-SLLM; rationale; limitations of TNM staging; references to existing approaches). |
|  | 3b | Reported — Introduction, paragraphs 2–3. Target population = CRC-SLLM patients; intended purpose = individualized OS prediction and risk-stratified selection for primary tumor resection; intended users = clinicians/surgeons in multidisciplinary care. |
|  | 3c | Not reported — health inequalities between sociodemographic groups were not specifically assessed. |
| Objectives | 4 | Reported — Introduction, final paragraph. States both development (SEER) and external validation (Chinese cohort). |
| **METHODS** | | |
| Data | 5a | Reported — Methods §2.1 (development: SEER 17 registries; evaluation: Fourth Hospital of Hebei Medical University), with rationale and representativeness. |
|  | 5b | Reported — Methods §2.1–§2.2 (diagnosis years 2010–2015; SEER 17 registries 2000–2022). End of follow-up date not explicitly stated; OS censored at last follow-up (§2.4). |
| Participants | 6a | Reported — Methods §2.1 (US population-based registry, 17 registries; single Chinese tertiary hospital). |
|  | 6b | Reported — Methods §2.2 (inclusion/exclusion criteria; identical for both cohorts). |
|  | 6c | Reported — Methods §2.3. Treatments (chemotherapy, PTR, metastasectomy) recorded and entered as model variables. |
| Data preparation | 7 | Reported — Methods §2.2–§2.3 (ICD-O-3 coding, variable extraction, missing-data handling). Similarity of preprocessing across sociodemographic groups not specifically evaluated. |
| Outcome | 8a | Reported — Methods §2.4. Outcome = overall survival (time from diagnosis to all-cause death/last follow-up); time horizons 12 and 24 months (§3.3). |
|  | 8b | Not applicable — OS is an objective endpoint (all-cause death) ascertained from registry/records; no subjective outcome assessment. |
|  | 8c | Not applicable — objective endpoint; blinding of outcome assessment not relevant. |
| Predictors | 9a | Reported — Methods §2.3 (14 predefined candidate clinicopathological variables). |
|  | 9b | Reported — Methods §2.3 (definitions and categorization of all predictors; consistent definitions across cohorts). |
|  | 9c | Not applicable — predictors are objective clinicopathological/registry variables; no subjective interpretation. |
| Sample size | 10 | Reported — Methods §2.2 and Results §3.1 describe how the cohort sizes were derived (16,703 retrieved → 1,153 eligible; 7:3 split; 65 external). No formal a priori sample-size calculation was performed. |
| Missing data | 11 | Reported — Methods §2.3. Variables with missing values (e.g., CEA) retained as a separate 'Unknown' category. |
| Analytical methods | 12a | Reported — Methods §2.2 (7:3 random partition into training/internal validation) and §2.4. |
|  | 12b | Reported — Methods §2.3–§2.4. Continuous variables categorized at clinically established cut-offs; predictors handled as categorical. |
|  | 12c | Reported — Methods §2.4. Univariate Cox screening → LASSO Cox selection → multivariable Cox; multicollinearity (VIF), PH assumption (Schoenfeld residuals); internal validation via the held-out internal cohort. |
|  | 12d | Not applicable — heterogeneity across clusters/centres not formally modelled (single external centre). |
|  | 12e | Reported — Methods §2.4. C-index and time-dependent AUC (discrimination), calibration plots (1,000 bootstraps), and decision curve analysis (clinical utility). |
|  | 12f | Not applicable — no model updating/recalibration was performed. |
|  | 12g | Reported — Results §3.3 (nomogram, Figure 2b) and §3.7 (publicly accessible web-based calculator: https://hbykdxdsyy.shinyapps.io/SLLM_Nom_Cox_model/). |
| Class imbalance | 13 | Not applicable — no class-imbalance handling methods were used. |
| Fairness | 14 | Not addressed — model fairness across sociodemographic groups was not specifically evaluated. |
| Model output | 15 | Reported — Results §3.3 (predicted 12- and 24-month OS probabilities); risk dichotomization threshold (prognostic score −0.291) defined in §3.7. |
| Training vs evaluation | 16 | Reported — Methods §2.1–§2.2 and Results §3.1 (identical eligibility, outcome, and predictors; differences in setting: US registry vs Chinese single institution), also addressed in Discussion §4. |
| Ethical approval | 17 | Reported — Methods §2.1 and 'Ethics approval and consent to participate'. SEER exempt (de-identified public data); external cohort approved by the Ethics Committee of the Fourth Hospital of Hebei Medical University (IRB 2026KY104-01). |
| **OPEN SCIENCE** | | |
| Funding | 18a | Reported — 'Funding' section (National Science and Technology Major Project 2024ZD0520305; Hebei Medical Science Research Project 20220142). |
| Conflicts of interest | 18b | Reported — 'Competing Interests' section (authors declare none). |
| Protocol | 18c | Not applicable — a study protocol was not prepared. (Recommend stating this explicitly in Methods.) |
| Registration | 18d | Not applicable — the study was not registered. (Recommend stating this explicitly.) |
| Data sharing | 18e | Reported — 'Data Availability' section (SEER publicly available; external de-identified data from corresponding author on reasonable request). |
| Code sharing | 18f | Partially — the implemented model is accessible via the web-based calculator (§3.7); the analytical code is not publicly deposited (available from the corresponding author on reasonable request). |
| **PATIENT & PUBLIC INVOLVEMENT** | | |
| Patient & Public Involvement | 19 | No patient or public involvement in the design, conduct, reporting, or dissemination of this study. |
| **RESULTS** | | |
| Participants | 20a | Reported — Results §3.1 and Figure 1 (participant flow: 16,703 → 1,153; cohort allocation). |
|  | 20b | Reported — Results §3.1 and Table 1 (baseline demographic, clinicopathological, and treatment characteristics for each cohort). |
|  | 20c | Reported — Table 1 presents the distribution of predictors and outcome across development and external-validation cohorts for comparison. |
| Model development | 21 | Reported — Results §3.1 (training n=808; internal validation n=345; external n=65). Number of outcome (death) events per cohort not explicitly tabulated — recommended addition. |
| Model specification | 22 | Reported — full model presented as the nomogram (Figure 2b, §3.3) and deployed for new-individual prediction via the freely accessible web calculator (§3.7). |
| Model performance | 23a | Reported — Results §3.4 (time-dependent AUCs with 95% CIs for all cohorts), §3.5 (calibration), §3.6 (DCA), and §3.7 (performance within low-/high-risk subgroups; KM with log-rank). |
|  | 23b | Not applicable — heterogeneity of performance across clusters not assessed. |
| Model updating | 24 | Not applicable — no model updating was performed or reported. |
| **DISCUSSION** | | |
| Interpretation | 25 | Reported — Discussion §4 (opening paragraphs): overall interpretation in the context of objectives and prior studies. |
| Limitations | 26 | Reported — Discussion §4 (exploratory-analysis paragraph) and Conclusion: small external cohort and PTR subgroups, retrospective design, selection bias, absence of molecular data. |
| Usability of the model | 27a | Partially — handling of missing/unavailable inputs addressed via the 'Unknown' category (§2.3); broader handling of poor-quality input data not otherwise specified. |
|  | 27b | Reported — Results §3.3 (step-by-step nomogram use by clinicians) and §3.7 (web calculator); expertise required = treating clinician. |
|  | 27c | Reported — Discussion §4 (final paragraph) and Conclusion: prospective multicentre validation of risk-stratified surgical strategy warranted. |

*Prepared to accompany manuscript submission. Items annotated "Not applicable" reflect that this is a conventional LASSO–Cox regression prognostic model rather than a machine-learning/AI or clustered model; the corresponding TRIPOD+AI items (fairness, class imbalance, cluster heterogeneity, model updating, subjective outcome/predictor assessment) do not apply.*
